# The Relationship Between Adult Attachment and Complicated Grief: A Systematic Review

**DOI:** 10.1177/00302228221083110

**Published:** 2022-05-29

**Authors:** Victoria Russ, Lusia Stopa, Katy Sivyer, Jane Hazeldine, Tess Maguire

**Affiliations:** 1 7423 University of Southampton, Southampton, Hampshire, UK; 2 427773 Mountbatten Hospice, Newport, Isle of Wight, UK

**Keywords:** Complicated grief, bereavement, attachment, loss, coping/adaptation

## Abstract

Attachment insecurity, including attachment anxiety and attachment avoidance, is proposed as a key factor disrupting adaptive recovery following bereavement, resulting in complicated grief. However, findings are inconsistent across studies. This review aimed to synthesise existing research on attachment patterns in adults experiencing complicated grief to better understand this relationship. 22 cross-sectional and longitudinal studies (5149 participants), published between 2003 and 2020, met inclusion criteria. Higher levels of attachment anxiety were consistently associated with symptoms of complicated grief. Higher levels of attachment avoidance were associated with symptoms of complicated grief, although this relationship was less consistent. The review has implications for clinical practice as bereaved adults with insecure attachment histories may be particularly vulnerable to experiencing complicated grief. The research is limited by the reliance on mainly cross-sectional studies. Future research should focus on longitudinal studies, and studies that explore men’s experiences, and of individuals living in non-Western countries.

## Introduction

### Complicated Grief

The death of a loved one is a highly stressful and painful event which is experienced uniquely by each individual (Shear, 2012). Initial reactions to a bereavement can include a range of thoughts, emotions and behaviours that often manifest as intense sadness and yearning, intrusive images and temporary loss of interest and engagement in activities (Shear, 2012). For most people, these experiences subside with time and they re-engage in activities, make meaning out of the loss, and integrate the loss into their ongoing life (Shear & Shair, 2005). However, for around 10–20% of individuals, the experience of intense grief extends beyond the time which is typically considered adaptive (around 6 months) and has a significant impact on functioning in daily life; this is conceptualised as ‘complicated grief’ (Shear & Shair, 2005).

Complicated grief is a persistent form of intense grief characterised by intrusive thoughts or images, a chronic sense of emptiness, difficulty accepting the painful reality of the death, intense yearning and sorrow and preoccupation with thoughts of the deceased (Boerner et al., 2013; Shear & Gribbin, 2016).

### An Attachment Perspective on Complicated Grief

Attachment theory has emerged as one of the primary paradigms for understanding adjustment to grief (Shaver & Fraley, 2008; Stroebe et al., 2005). The loss of a loved one through death is an event that triggers activation of the attachment system, giving rise to emotional and behavioural responses that serve to relieve distress through seeking proximity to others. An attachment theory view on adaptive, ‘normative’ bereavement centres on the premise that the death of a loved one, that is, an attachment figure, will trigger predictable responses for most people such as strong protest, anger, yearning, despair, intense sorrow, loneliness and withdrawal. Over time, however, individuals gradually adjust to the loss by maintaining a symbolic relationship with their deceased loved one, restoring their sense of security and, and re-engaging with a new reality (Bowlby, 1980).

Early conceptualisations of adult attachment orientations used categorical models to classify individuals into a single attachment style. These categories have been referred to as secure, fearful, dismissing, and preoccupied (Bartholomew & Horowitz, 1991). More recently, however, researchers have found that categorical models lack reliability and validity, and a dimensional approach has been more widely adopted in which attachment is measured along two continuums of attachment anxiety and attachment avoidance (Fraley & Shaver, 2000). Attachment anxiety refers to the extent to which individuals worry that their partners will not be available at times of need, and have a tendency to fear rejection and abandonment (Mikulincer & Shaver, 2007). Attachment avoidance refers to the extent to which individuals seek to maintain autonomy and emotional distance from relationship partners, and have a tendency to lack trust in others (Fraley & Shaver, 2000).

Bowlby (1980) proposed that attachment insecurities can complicate the grief process. According to Bowlby, high levels of attachment anxiety may predict ‘chronic mourning’ which is characterised by overwhelming anxiety and sadness, prolonged difficulty in re-engaging with adaptive functioning and forming new relationships, preoccupation with the deceased, and difficulty accepting the loss. Anxiously attached individuals tend to experience chronic activation of the attachment system, leading to hyper-accessibility of thoughts of the deceased loved one which may perpetuate excessive yearning (Mancini & Bonanno, 2012).

Attachment avoidance, on the other hand, is proposed to underlie ‘delayed grief’ whereby attachment-related thoughts and emotions are suppressed and urges to seek support are inhibited (Mikulincer & Shaver, 2007). Individuals high in attachment avoidance are thought to respond to grief with a de-activation of their attachment system, leading to a loss of access to thoughts and images of lost loved ones (Mikulincer et al., 2002). Attempts to suppress painful thoughts following a bereavement are likely to fail to reduce distress in the long term however, and suppressed pain may resurface when cognitive or emotional demands increase (Berant et al., 2008).

### Current Review

Despite attachment theory being a key paradigm for understanding individual differences in reactions to typical experiences of bereavement, there have been limited attempts to systematically summarise and evaluate the literature on the relationship between attachment insecurity and complicated grief. To our knowledge, there are just two existing systematic reviews that have explored risk factors for complicated grief that include attachment styles (Lobb et al., 2010; Mason et al., 2020).

Although both reviews supported a relationship between insecure attachment and complicated grief, each had limitations. A key issue with the Lobb et al. (2010) review was that it did not distinguish between different insecure attachment dimensions, which is not in line with current conceptualisations of attachment. Several studies have also been published since this review so this evidence-base needs updating. The Mason et al. review, although very recent, was constrained to papers published in English in North America between 2008 and 2018, and focused only on bereaved caregivers. Consequently, the Mason et al. (2020) review based its conclusions about attachment style and complicated grief on only two studies, one of which was exploring attachment insecurity as a predictor of psychotherapy outcome, rather than complicated grief symptoms specifically, and therefore misses several studies in this area.

Furthermore, although empirical evidence has largely supported the proposal that attachment anxiety has a significant association with complicated grief reactions (e.g., Boelen & Klugkist, 2011; Currier et al., 2015; Field & Filanosky., 2010; Mancini et al., 2009; Meier et al., 2013), there are studies which do not report such an association (Berenguer-Perez et al., 2018). The literature regarding attachment avoidance and grief outcome is even less consistent and has yielded conflicting results (Mikulincer & Shaver, 2016). There is therefore a need to systematically and specifically examine the literature on attachment and complicated grief to better understand these relationships across a range of bereavement types and contexts, for example, cause of death, relationship to deceased, suddenness of death and ethnicity.

The aim of the current systematic review is to thoroughly examine and synthesise the evidence regarding attachment-related anxiety and avoidance and its relationship to complicated grief. It aims to extend previous reviews by including studies published in any language, with no limitations on country or date range. It will also examine grey literature to ensure full coverage of the literature available. Given the inconsistencies in previous findings regarding the relationship between attachment insecurity and complicated grief, the review will also consider the empirical evidence of potential mediators and moderators of this relationship.

## Aims

This systematic review was designed to answer the following questions:

1. Are higher levels of attachment anxiety and attachment avoidance associated with elevated symptoms of complicated grief?
2. What factors may mediate or moderate these proposed relationships?
3. What is the quality of the available empirical evidence and how does this impact our ability to draw reliable conclusions from the literature?

## Method

### Search Strategy

The protocol for this systematic review was published on Prospero (Prospero ID: CRD42019145677). Five electronic databases relevant to psychological research were searched in December 2020 (PsycINFO, MEDLINE, Web of Science, Health and Social Care Evidence Search, Cochrane Library). Table 1 shows the search terms and syntax that were used for the search strategy. The search strategy contained no limits, including date of publication or original language of article. Inclusion and exclusion criteria can be found in Table 2.

**Table 1.** Search terms and syntax for systematic review.

	Attachment	Complicated (grief)	Grief
*Search terms*	“Parent* N2 attachment” OR “parent” bond*” OR “early OR first N1 relationship” OR “attachment behavio?r”	complicat* OR traumatic* OR prolong* OR persist?nt* OR abnormal OR “persistent complex grief” OR “persistent complex bereavement”	Grief OR griev* OR bereavement OR mourn*

**Table 2.** Eligibility Criteria.

	Inclusion	Exclusion
Types of study and publication type	Published and unpublished empirical studies. All study designs.	Conference posters, abstracts, reviews and proposals.
Participants	Participants were adults, that is, 18 years and over. Participants had experienced a bereavement through the death of a human (not a pet)	Participants whose loss was characterised as non-death, that is, job loss, relationship breakdown.
Focus of study	The link between attachment insecurity and pathological grief was explored empirically.	Due to the limited scope of the review, and to increase homogeneity, studies were excluded if the nature of the death was miscarriage.
Outcomes	A measure of attachment was included as a primary or secondary outcome in the study. A measure of complicated, prolonged, pathological or traumatic grief was included as a primary or secondary outcome in the study.	Studies that measured typical responses to bereavement only (rather than complicated, pathological, prolonged or traumatic grief).

### Study Selection

**Figure 1.**
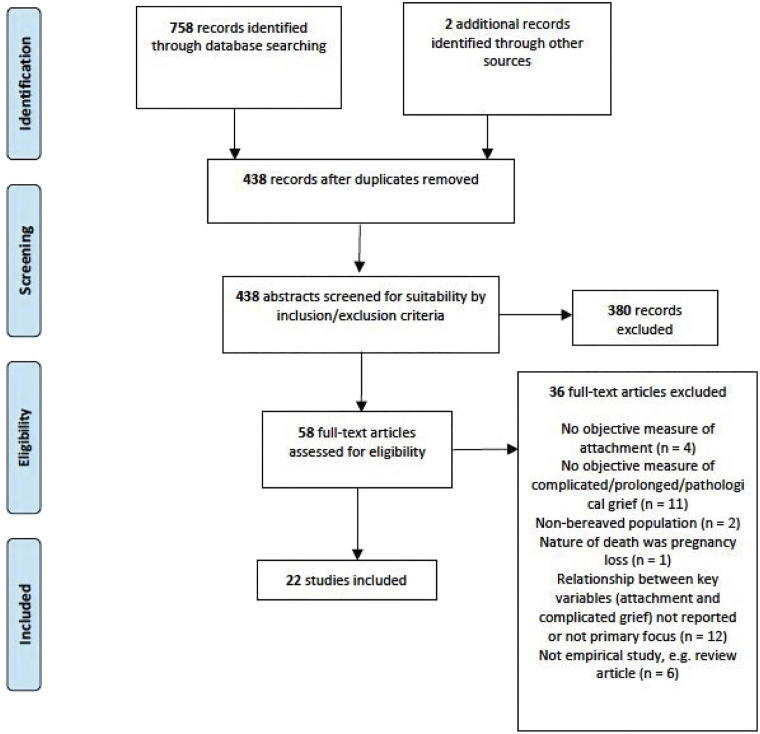
PRISMA diagram for systematic review of literature.

Citations were collated into a referencing software package (EndNote) and duplicates were removed. Additional studies were identified through searching reference lists. All remaining records were screened by their title and abstract against inclusion and exclusion criteria (Table 2) to determine eligibility for review. After excluding unsuitable papers, the full-text versions of remaining papers were obtained and further screened against the eligibility criteria. Articles written in a language other than English were translated by colleagues within the department (i.e., Berenguer-Perez et al., 2018). From this screening stage, eligible papers were identified to be included in the final synthesis (see Figure 1).

### Data Extraction

Relevant information from each of the final studies was extracted to address the primary aims of the review and provide context to the study and participants. Data was then synthesised using a narrative approach (Popay et al., 2006).

### Quality Assessment

The quality of the final selected studies was assessed using the QualSyst tool, which can be used for evaluating quantitative studies (Kmet et al., 2004). Each of the 14 standards are rated on a 0–2 scale (0 = standard not met; 1 = partially met; 2 = standard met). The total score for each paper is calculated as a percentage of the total possible score. Quality assessment was undertaken by two independent raters to increase reliability (VR and IC). Initial inter-rater agreement was calculated at 67%. Where overall scores were different, agreement was reached through reviewing and discussing the paper together.

## Results

### Search

The present review identified 22 studies exploring the relationship between attachment style and complicated grief symptoms that met inclusion criteria. Table 3 summarises the extracted data and includes: aims of the study, location of research (country), sample characteristics, design, key outcome measures and key statistical findings, including exploration of potential moderating or mediating factors. Quality assessment scores are also presented in Table 3.

**Table 3.** Summary of studies selected for review.

Study Reference	Location	Sample	Design	Measures	Key findings and significance values	Quality Assessment Score (%)
Berenguer-Perez et al. (2018) ^i^	Spain *(translated from Spanish to English)*	50 bereaved psychology undergraduate students (68% female). Age = 20–40 years (M = 22.97 years).	Cross-sectional	El cuestionario de apego adulto (adult attachment questionnaire); inventory of complicated grief (Spanish version)	No significant association between attachment style (secure [*r* = −0.13]., anxious [*r =* 0.20], or avoidant [*r* = −0.10]) and CG; all *p*s > .17, ns;	82
Boelen and Klugkist (2011)	The Netherlands	348 bereaved adults recruited from general population (90.5% female). Age = 18–74 years (M = 42.4 years).	Cross-sectional	Inventory of complicated grief; relationship questionnaire	Attachment anxiety (*B* = 0.11) not associated with PGD (*p*>.05, ns). Attachment avoidance (*B* = 0.45) significantly associated with PGD, (*B* = .69, *p<*.05;).	91
Campisano-Baugnon (2004)	USA	75 bereaved adults (64% female) whose partner had died 3–6 months prior. Age = 21–55 years (M = 45.1 years)	Cross-sectional, correlational survey	Texas revised inventory of grief; reciprocal attachment questionnaire	Insecure adult attachment patterns (compulsive self-reliance [*r* = −0.09*]*; compulsive care-seeking [*r =* 0.18]; angry withdrawal [r = 0.02]) not significantly associated with CG, all *p*s >.05, ns.	86
Currier et al. (2015)	USA	195 students, bereaved in the past 2 years (80% female). Age = 18–49 years (M = 21 years).	Cross-sectional	Experiences in close relationships - relationship structures; inventory of complicated grief – revised	Attachment avoidance and attachment anxiety associated with higher levels of CG (*B* = 0.14, *p*<.05; *B* = 0.19, *p*<.01 respectively).	91
Delespaux et al. (2013)	Belgium	321 bereaved adults recruited from general population (89% female) who had experienced death of a romantic partner. Age = 17–88 years (M = 41 years).	Cross-sectional	Experiences in close relationships; inventory of traumatic grief (French version).	Avoidant attachment associated with grief severity (*r* = 0.19, *p*<.01). Anxious attachment associated with grief severity (*r* = −.021, *p*<.01).	91
Edelson (2009)	USA	402 bereaved adults (94% female). Age = 18–78 years (M = 49.34 years)	Cross-sectional, correlational survey	Relationship questionnaire; relationship scales questionnaire; prolonged grief Disorder-13	Participants with fearful (OR = 4.01, *p*<.001), preoccupied (OR = 4.07, *p*< .05) or dismissing (OR = 4.59, *p*< .001) attachment styles more likely to be classified with PGD. Securely attached participants were less likely to screen positively for PGD (*F* = 12.85, *p*<.001). Participants with PGD were more likely to report higher levels of anxious (*F* = 24.43, *p<* .001 and avoidant attachment (*F =* 32.27, *p* < .001).	95
Field & Filanosky (2009)	USA	502 bereaved adults recruited from general population (84% female). Mean age = 34.45 years (range not reported).	Cross-sectional	Relationship questionnaire; inventory of complicated grief – Screen	Anxious attachment and avoidant attachment positively associated with CG when controlling for time since death and relationship closeness (anxious, *r* = 0.21, *p*<.001; avoidant, *r = 0.18, p* <.001 ).	86
Gegieckaite and Kazlauskas (2020)	Lithuania	203 bereaved adults recruited from general population (85.2% female). Mean age = 42.13 years (range = 19–92).	Cross-sectional	Experiences in close relationships – Relationship structure questionnaire; Prolonged grief Disorder-13	Anxious attachment significantly positively associated with prolonged grief, *B* = .16, *p* < .01; avoidant attachment significantly positively associated with prolonged grief, *B* = .24, *p* < .01. However, effect of anxious attachment was not significant when emotion regulation difficulties were included.	95
Hyu, Kim, Lee, & Chae (2017)	Korea	81 bereaved parents (54.3% female) recruited from survivors of major ferry disaster. Mean age = 47.96 years (range not reported).	Cross-sectional	Experiences in close relationships questionnaire - short form; inventory of complicated grief	Avoidant attachment associated with CG (*r* = 0.26, *p*<.05). Attachment anxiety not associated with CG (*r* = 0.11, *p >* .05, ns).	82
Jerga et al. (2011)	USA	368 bereaved undergraduate students (67% female). Age = 17–49 years (M = 19.6 years).	Cross-sectional	Experiences in close relationships scale; experiences in close relationships - short form; attachment Network questionnaire; prolonged grief disorder – 13	General and specific attachment anxiety, and general avoidant attachment associated with prolonged grief (general anxiety, *B* = .12, *p* <.01; specific anxiety, *B* = .13, *p* <.01; general avoidance, *B* = .22, *p* < .01). Specific avoidance not associated prolonged grief (*r* = −0.03, *p* > .05, ns).	86
Levi-Belz and Levi-Ari (2019)	Israel	156 adults bereaved through suicide (81.4% female). Age = 18–70 years (M = 40.7 years).	Cross-sectional	Relationships questionnaire; inventory of complicated grief	Secure attachment style was negatively correlated with CG (*r* = −0.22, *p*<.01). Disorganised, avoidant and anxious attachment not significantly associated with CG (*r* = 0.13; *r* = 0.13; *r* = 0.02 respectively; all *p*s > .05, ns).	91
Maccallum and Bryant (2018)	Australia	285 bereaved adults (79.1% female) recruited from general population. Mean age = 48.89 years (range not reported).	Cross-sectional	Prolonged grief Disorder-13; experiences in close relationships	Attachment anxiety and avoidance significant predictor of membership to group with high levels of PGD (attach anxiety, *B* = −1.58, *p*<.001; attach avoid, *B* = −0.73, *p* < .001).	82
Mancini et al. (2009)	USA	50 conjugally bereaved adults (62% female). Mean age = 51.81 years (range not reported).	Longitudinal - 4 month and 18 month post-loss.	Structured clinical interview for DSM-III-R for PTSD and complicated grief (CG); relationship scales questionnaire	Avoidant and anxious attachment patterns significantly associated with CG symptoms at 4 months (*r* = 0.40, *p* <.01 [avoidant]; *r =* 0.44, *p* < .01 [anxious]) and 18 months (*r* = 0.30, *p* <.05 [avoidant]; r = 0.43, *p* < .01 [anxious]).	82
Meert et al. (2010)	USA	261 bereaved parents (69% female) whose child died in a paediatric intensive care unit 6 months earlier. Mean age = 37.2 years (range not reported).	Cross-sectional	Inventory of complicated grief; relationship scales questionnaire	CG associated with attachment-related anxiety (*r* = 0.47, *p*<.001) and attachment-related avoidance (*r =* 0.37, *p*<.001).	86
Meert et al. (2011)	USA	138 bereaved parents whose child died in a paediatric intensive care unit 18 months earlier (72% female). Age range not reported (*M* = 38.0 years).	Longitudinal – 6 month and 18 month post-loss.	Inventory of complicated grief; relationship scales questionnaire	Attachment-related anxiety and attachment-related avoidance was not associated with improvement in CG symptoms (*r* = 0.08, *p* = .27 [anxiety]; *r* = 0.30, *p* = .79 [avoidance])	86
Meier et al. (2013)	USA	656 undergraduate students bereaved within the last 2 years (81.4% female). *M* = 21.67 years (range not reported).	Cross-sectional	Experiences in close relationships - relationship structures questionnaire; inventory of complicated grief	Attachment anxiety associated with PGD (*B* = 0.30, *p*<.001). Attachment avoidance not associated with PGD (*B* = 0.05, *p*>.05).	91
Milman et al. (2019)	North America and Europe	171 adults bereaved within last 2–12 months (71.9% female). Age = 18–90 years; *M* = 44.30 years).	Longitudinal, 7–10 month follow-up	Experiences in close relationships – short form; prolonged grief Disorder-13;	Anxious and avoidant attachment associated with PGD (*r* = 0.22, *p*<.01; *r* = 0.48, *p* < .01).	91
Takacs (2008)	USA	124 conjugally bereaved adults (58.9% female). Age = 26–86 years (M = 49.82).	Cross-sectional, survey	Inventory of traumatic grief; relationship questionnaire	Insecure attachment style (OR = 23.05, *p*<.01), particularly fearful attachment style (OR = 1.93, *p*=.005), significant predictors of traumatic grief.	91
Van der Houwen, Stroebe, Stroebe, Schut, van der Bout & Wijngaards-de Meij (2010)	The Netherlands	195 adults who experienced the death of first-degree relative within the last 3 years (92.3% female). Age = 19–79 years (M = 41.50 years).	Longitudinal (baseline, 3 months, 6 months)	Experiences in close relationships - revised; DSM-V criteria for complicated grief	Attachment avoidance predicted CG (*B* = 0.06, *p* < .001). Attachment anxiety not a significant predictor (*B* = 0.03, *p* > .05).	82
Xu et al. (2015)	China	240 bereaved adults (56.2% female) of whom a family member died due to cancer mean age = 39.52 (range not reported).	Cross-sectional	Prolonged grief questionnaire – 13; experiences in close relationships	Attachment avoidance associated with CG (*r* = 0.22, *p* < .001). Attachment anxiety associated with grief (*r* = 0.32; *p* < .001).	95
Yu et al. (2016)	China	247 bereaved individuals (58.3% female). Age = 18–80 (M = 39.14 years).	Cross-sectional	Experiences in close relationships (Chinese version); prolonged grief questionnaire	Attachment anxiety associated with CG (*r* = 0.33, *p*<.001). Attachment avoidance associated with CG (*r* = 0.20, *p* < .01).	91

*Note:* CG = Complicated Grief; PGD = Prolonged Grief Disorder.

### Quality Assessment of Studies

All studies were of relatively high quality, scoring 82%–95% on the QualSyst tool (see Table 3). However, the method of participant recruitment and selection was an area of concern for many studies (11 out of 22), mainly due to the use of student populations or younger cohorts, which are unlikely to be representative of the target population. In addition, all the papers reported a self-selecting sample, which may bias results. The lack of clarity in how potentially confounding variables were controlled for within the analysis of data was also a problematic area across many studies.

### Study Characteristics

The majority of studies (*n =* 19) were published in peer-reviewed journals, and the remaining three studies were unpublished doctoral theses (Campisano-Baugnon, 2004; Edelson, 2009; Takacs, 2008). The studies were conducted in various countries and represented Northern, Eastern, Southern and Western hemispheres across the world, although most were conducted in developed countries. There were no studies from the UK. The higher numbers of studies carried out in the USA and The Netherlands is partly explained by experts in grief research being based in these countries; hence some researchers or research groups account for several of the final papers, for example Neimeyer (in Meier, Carr & Currier, 2013; Currier, Irish, Neimeyer, & Foster, 2015; Milman et al., 2019) in the USA, and Stroebe et al. in the Netherlands (Wijngaards-de Meij, et al., 2007).

The majority of studies (*n* = 17) utilised a cross-sectional design that analysed data from a bereaved population at a specific point in time, either within a restricted timeframe (e.g., within 3 years of bereavement), or with no restrictions on time since loss. Only five studies utilised a longitudinal design whereby data was collected at multiple time points to measure changes in grief symptomatology (e.g., four and 18 months post-loss). None of the studies utilised an experimental design, for example, using an attachment-priming method which activates cognitive representations of attachment security (e.g., Carnelly & Rowe, 2007).

### Participant Characteristics

5149 participants were represented collectively in the final papers.^1^ The mean sample size across the final studies was 235 (range = 50–656). The mean age of participants was 43.16 years, ranging from 17–92 years. Most studies recruited from the general population, or specific bereavement populations (e.g., parents of children who died in intensive care); however, four studies recruited solely from student populations, hence the cohorts of participants in these studies is much younger in comparison.

Across the 21 studies that reported ratios of gender representation, females accounted for a mean of 71.4% of the participants (range = 54.3%–94.0%), with all studies reporting a higher ratio of females taking part than males.

### Bereavement Experiences

The reviewed studies reflected a range of bereavement experiences, relating to the relationship with the deceased, the nature of the death, and the time elapsed since loss. While most papers reported on a variety of relationships, some studies focused on specific relationships, that is, bereaved through death of their partner (Delespaux et al., 2013; Mancini et al., 2009; Takacs, 2008) or death of their child (Huh et al., 2017; Meert et al., 2010, 2011; Wijngaards-de Meij et al., 2007). In studies that utilised a student population, participants most commonly reported losing a grandparent (Jerga et al., 2011), whereas studies that recruited from the general population tended to be grieving the loss of a partner or parent. These differences are likely to be reflective of the grief experiences at different life stages of students versus typical adult populations. Most studies represented a range of causes of death, for example, illness, accident, murder and suicide. Illness was the most common cause of death in all studies, which is reflective of the most common causes of death globally (World Health Organisation, 2016).

Collectively, the studies reflected a breadth of time since loss (range = 6 days–30 years), and so recall reliability is likely to vary between those who are more recently bereaved compared to those whose bereavement occurred decades previously. However, several studies recruited participants specifically based on their bereavement being within a certain timeframe (e.g., within past 2–12 months, Milman et al., 2019), which may increase the reliability of how participants recall their experiences at the time of their bereavement. Furthermore, the criteria for complicated grief states that a diagnosis cannot be made within the first 6 months of loss, reflecting the variety of ways that ‘typical’ grief is expressed in the first few months, giving a rationale for excluding participants who have experienced a very recent bereavement. Despite this exclusion criteria, some studies included individuals who had been bereaved less than 6 months previously (e.g., Delespaux et al., 2013; Levi-Belz & Levi-Ari, 2019), and this calls into question whether their experiences were truly representing complicated grief versus typical bereavement-related distress.

### Evidence of Association between Attachment Style and Complicated Grief

#### Categorical models

##### Secure attachment

Three studies investigated the relationship between the ‘secure’ category of attachment with complicated grief (Table 4). Two of these reported consistent evidence for an association between secure attachment and lower levels of complicated grief (Levi-Belz & Levi-Ari, 2019), which is in line with expectations from an attachment perspective on grief. This association was observed in response to various causes of bereavement, for example, illness, accident, murder (Edelson, 2009) or suicide (Levi-Belz & Levi-Ari, 2019). Furthermore, this association was found across various relationships to the bereaved, that is, child, partner, parent, and across a wide range of ages (18–78) and held across two countries (USA and Israel). However, the samples in both studies were mainly female and it is not clear whether the findings also generalise to males.

**Table 4.** Overview of Evidence Regarding Association Between Attachment Style and Complicated Grief.

Attach style	Positive association with complicated grief				No or negative association with complicated grief			
Secure					Berenguer-Perez et al. (2018) Edelson (2009) (Negative) Levi-Belz and Levi-Ari (2019) (Negative)			
Insecure	Takacs (2008)							
	**Anxious**	Boelen and Klugkist (2011) Delespaux et al. (2013) Edelson (2009) Field and Filanosky (2010) Gegieckaite and Kazlauskas (2020) Jerga et al. (2011) Mancini et al. (2009) Meert et al. (2010, 2011) Meier et al. (2013) Milman et al. (2019) Van der Houwen et al. (2010) Xu et al. (2015) Yu et al. (2016)	**Preoccupied**	Edelson (2009) Takacs (2008)	**Anxious**	Berenguer-Perez et al. (2018)	**Preoccupied**	Campisano baugnon (2004) Levi-Belz and Levi-Ari (2019)
			**Fearful/ disorganised**	Edelson (2009) Takacs (2008)			**Fearful/disorganised**	Levi-Belz and Levi-Ari (2019)
	**Avoidant**	Boelen and Klugkist (2011) Edelson (2009) Field and Filanosky (2010) Gegieckaite and Kazlauskas (2020) Huh et al. (2017) Jerga et al. (2011) Mancini et al. (2009) Meert et al. (2010; 2011) Meier et al. (2013) Milman et al. (2019) Van der Houwen et al. (2010) Wijngaards-de Meij et al., (2007) Xu et al. (2015) Yu et al. (2016)			**Avoidant**	Berenguer-Perez et al. (2018) Delespaux et al. (2013) **(negative)**		
			**Dismissing**	Edelson (2009) Takacs (2008)			**Dismissing**	Campisano-Baugnon, 2004 Levi-Belz and Levi-Ari (2019)

In contrast, one of the three studies did not find any association between secure attachment style and complicated grief (Berenguer-Perez, et al., 2018). However, the reliability of this study may be questionable, as the quality assessment was lower than the other two studies, and the sample size was relatively small (*N* = 50), Hence, it may not have been sufficiently powered to detect statistically significant results.

##### Insecure Attachment

One study in the review used the single broad category of insecure attachment style, and reported a significant positive association with complicated grief (Takacs, 2008; Table 4). This study included 124 bereaved adults whose partners had most commonly died through illness and those with an insecure attachment style were over 23 times more likely to meet criteria for complicated grief than those who had a secure attachment style.

A further four studies used the three-category model of insecure attachment (preoccupied; fearful/disorganised; dismissing) but the findings were inconsistent regarding the relationship with complicated grief (Table 4). However, this inconsistency may be accounted for by the limited reliability of using categorical approaches to understand attachment. Two of these studies (Edelson, 2009; Takacs, 2008) reported a positive association with all three insecure attachment styles and complicated grief. This association was most prevalent for the fearful attachment style (characterised by high avoidance and high anxiety). Both of these studies are unpublished doctoral theses which may warrant caution when interpreting the findings as they have not undergone peer-review; however, both studies were of high quality achieving scores of over 90% in the quality assessment. The other two studies (Campisano-Baugnon, 2004; Levi-Belz & Levi-Ari, 2019) did not find any significant association between the insecure attachment categories and complicated grief. However, these two studies may be less reliable than others given the small sample size in Campisano Baugnon (2004), and the fact that bereavement was within the past three to 6 months, which means the measure of complicated grief is unlikely to be valid. Furthermore, the other study by Levi-Belz and Levi-Ari (2019) was focused on survivors of suicide loss and the findings may therefore be specific to this population, rather than generalisable across other forms of loss.

#### Dimensional Models

##### Attachment anxiety

15 studies investigated the relationship between attachment anxiety and complicated grief using dimensional measures (Table 4). This included nine cross-sectional studies and five longitudinal studies. Across the cross-sectional studies, higher levels of attachment anxiety were consistently associated with increased levels of complicated grief. 14 studies found a significant association between higher levels of attachment anxiety and elevated symptoms of complicated grief. This finding was consistent despite heterogeneity in study and sample characteristics (i.e., age, study design, measures used), and nature of bereavement (nature of death, relationship to deceased, time since loss). There was only one cross-sectional study that did not find any significant association between attachment anxiety and complicated grief (Berenguer-Perez et al., 2018). This study recruited 50 undergraduate students across Spain. Most individuals in the study (54%) had experienced the death of a grandparent or aunt/uncle (22%) and therefore the failure to find significant results may reflect the fact that the bereavements may have been less impactful compared with studies where participants had lost a first-degree relative such as a child, parent or spouse. This study yielded lower quality scores in comparison to the other studies (82%), particularly due to its’ relatively small sample size, and hence, it may have lacked sufficient power to detect statistically significant results. This study was also the only one conducted in Spain, so it is possible that cultural differences in grief expression may partly account for the contrasting finding.

Four of the five longitudinal studies identified attachment anxiety as a significant predictor of complicated grief (Mancini et al., 2009; Meert et al., 2011; Milman et al., 2019; Wijngaards-de Meij et al., 2007; Table 4), a relationship which was held at 18 months post-loss in both the Mancini et al. (2009) and Meert et al. (2011) study. In contrast, Van der Houwen and colleagues (2010) found that the effect of attachment anxiety on complicated grief was no longer significant when it was examined together with social support and emotional loneliness. The authors conclude that it is important to consider the pathways between factors to understand their interaction, and also consider including bereavement-specific and general outcome measures to better understand the experience of the emotional impact of bereavement.

##### Attachment avoidance

17 studies investigated the relationship between attachment avoidance and complicated grief (Table 4). 12 of these studies utilised a cross-sectional design, and five used a longitudinal design. The evidence for an association between higher levels of attachment avoidance and higher levels of complicated grief was relatively consistent. Of the 12 cross-sectional studies, 10 studies reported a positive association between attachment avoidance and complicated grief. This relationship was consistent across a wide age range of participants (18–92 years), various countries (China, Korea, the Netherlands, USA, Lithuania), and bereavement experiences (e.g., death by illness, accident, murder, suicide). Two of the cross-sectional studies reported no significant association; however, one study may have lacked sufficient power to detect significant results (Berenguer-Perez et al., 2018).

All five longitudinal studies consistently found that attachment avoidance was a significant predictor of complicated grief symptoms when measured between four and 20 months later (Table 4). Meert and colleagues (2011) further reported that attachment-related avoidance was associated with less improvement in complicated grief symptoms over time and attachment avoidance was highlighted as a risk factor for persistent grief related distress.

### Mediating and Moderating Factors in the Relationship between Attachment Style and Complicated Grief

#### Potential mediators

Four studies in the review explored potential mediating factors in the relationship between attachment and complicated grief. Three studies used a cross-sectional design (Gegieckaite & Kazlauskas, 2020; Milman et al., 2019; Yu et al., 2016) and one used a longitudinal design (Boelen & Klugkist, 2011). All studies examined coping strategies as potential mediators.

Externalised forms of continuing bonds, for example, an ongoing inner relationship with the deceased that involves hallucinations was identified as a mediator by Yu et al. (2016). In this study, individuals high in attachment anxiety and attachment avoidance were more likely to cope through using externalised continuing bonds, which in turn predicted elevated grief symptoms (Yu et al., 2016).

In their study of 203 bereaved adults, Gegieckaite and Kazlauskas (2020) identified that emotion regulation difficulties mediated the link between anxious attachment and prolonged grief, and partially mediated the link with avoidant attachment. In addition, Boelen and Klugkist (2011) and Milman et al. (2019) identified that cognitive processes i.e., high levels of rumination, catastrophic misinterpretations about grief, and negative thoughts about the future acted as significant mediators between higher attachment anxiety and avoidance, and complicated grief symptoms. In addition, individuals high in attachment anxiety and avoidance were more likely to cope by using avoidance strategies which mediated the relationship with higher level of complicated grief symptoms (Boelen & Klugkist, 2011).

These are the only studies to examine potential mediators in the relationship between attachment and complicated grief, and therefore to make more firm conclusions, further research is warranted to replicate these findings. In addition, the current evidence base is heavily drawn from cross-sectional research which cannot establish causality, hence more longitudinal studies are needed.

#### Potential moderators

Higher levels of self-disclosure, and lower use of continuing bonds, i.e., a continued symbolic relationship to the deceased, (in individuals high in attachment anxiety only) were found to weaken the relationship between attachment insecurity and complicated grief (Currier et al., 2015; Levi-Belz & Levi-Ari, 2019). However, the use of continuing bonds strategies as a moderator has received mixed findings, and therefore it is not clear whether these strategies are adaptive or maladaptive in coping with grief in the context of different attachment styles (Field & Filanosky, 2010).

Relationship factors are important in understanding how attachment styles may interact differently with grief response. Level of conflict in the relationship with the person who died may moderate the link between attachment insecurity and complicated grief (Jerga et al., 2011). Jerga et al. (2011) found that individuals with high attachment anxiety and high levels of conflict scored higher on complicated grief than individuals with low attachment anxiety who had high levels of conflict. Mancini et al. (2009) reported that high marital quality predicted lower levels of grief only in individuals with a dismissing avoidant style (high avoidance, low anxiety). In the other three categories of attachment (preoccupied, fearful and secure) high marital quality predicted increased grief symptoms. These studies provide preliminary evidence that it may be important to consider how an individual’s attachment history may interact with other key factors in predicting response to grief.

## Discussion

The aim of this systematic review was to examine and synthesise the evidence regarding attachment-related anxiety and avoidance, its relationship to complicated grief across different types of bereavement (e.g., nature of death, relation to deceased) and across a range of contexts (e.g., different national and cultural groups), and to consider potential mediators and moderators of this relationship. This review consolidated a mixture of 22 cross-sectional and longitudinal studies, which were generally all deemed to be of high-quality, and which provided representation of a wide range of bereavement types and contexts, e.g., various causes of death, range of suddenness of death, variety of relationship types to deceased. Overall, the findings suggest that higher levels of attachment anxiety and attachment avoidance are both positively associated with complicated grief symptoms. In particular, longitudinal studies supported the notion that attachment insecurity (anxiety and avoidance) is predictive of complicated grief up to 20 months post-loss. By addressing limitations of prior reviews, this systematic review offers an updated, more focused synthesis of the evidence-base by including an additional 21 studies that have not been considered in similar reviews (Lobb et al., 2010; Mason et al., 2020). This is also the first systematic review to distinguish between different attachment dimensions (anxiety and avoidance) and therefore brings the evidence base in line with current conceptualisations of attachment and deepens our understanding of the relationship between attachment and complicated grief.

The findings of this review are consistent with theoretical formulations by Bowlby (1980) who stated that the way people manage their grief can be understood as a function of their attachment histories. Bowlby proposed that individuals with insecure attachment orientations, compared with secure, are likely to experience complications in the grieving process as the lack of security in relationships interferes with the ability to adaptively seek safety and comfort in others. In attachment theory, the relationship between attachment anxiety and higher levels of grief is explained by hyper-activation of the attachment system whereby the bereaved individual is likely to regulate their emotions by signalling or expressing their needs and fears, exaggerating their distress and presenting themselves as extremely vulnerable to pain (Shaver & Mikulincer, 2002).

The findings of this review also suggest a relatively clear association between avoidant attachment and complicated grief across various types of bereavement contexts and across a range of countries. According to attachment theory, individuals with an avoidant orientation are likely to deal with distress and threat by deactivating their attachment system, forgoing support seeking, and relying on themselves to deal with threats (Mikulincer & Shaver, 2016). Although some theorists have proposed that avoidance may sometimes be adaptive, offering a buffer to overwhelming emotions, the findings of this review would suggest that, in the long run, attachment-related avoidance may be linked with complicated grief. Previous research has demonstrated the fragility of avoidant defences, particularly when cognitive or emotional load increases (e.g., Fraley & Shaver, 1997; Kohn et al., 2012).

### Strengths and Limitations

The studies were all deemed to be high-quality research and most studies utilised well-validated measures that had good psychometric properties. Strength of this review is the inclusion of unpublished research and studies written in any language. These inclusive factors are likely to have reduced the risk of publication bias and have yielded a more representative examination of the literature.

A limitation across all studies was the underrepresentation of males. Although this is a consistent problem in grief research, if we are to make confident conclusions regarding the way that men experience grief, there need to be more concerted efforts to hear the voices of bereaved men.

The evidence base consisted mostly of cross-sectional studies hence conclusions regarding the direction of the effect cannot be made. All studies were retrospective and the experience of bereavement may have affected how individuals perceived and reported their attachment historie (Davila & Cobb, 2003). However, longitudinal studies were included as part of this review and their findings were consistent with the cross-sectional findings. Further longitudinal studies that measure attachment prior to a bereavement would allow methodological limitations to be addressed, and provide further clarity regarding the direction of the relationship. While the studies that explored mediators and moderators may help to build a more nuanced understanding of the relationship between attachment insecurity and complicated grief, very few studies explored these factors and none of the findings have been replicated. Further research in this area is needed.

Most studies included in the review were conducted in the USA, with a lack of representation from Africa, South America and the UK. Hence, caution is warranted when considering how the findings generalise across cultures and ethnicities. A meta-analysis may have enabled a better understanding of the differences between studies; however, it was not appropriate for this review due to the heterogeneous nature of the measures of attachment and complicated grief, and the heterogeneity among samples. Future research could focus on conducting a meta-analysis on studies that share similarities, for example, those utilising one particular measure. In addition, screening of the literature was undertaken by one reviewer and so reliability of applying the eligibility criteria is not known.

### Implications

Attachment-related insecurity may act as a potential vulnerability factor following a bereavement. Therapeutic interventions to support individuals experiencing complicated grief may benefit from considering their attachment orientations as individuals with anxious or avoidant styles may require support in different ways. According to a meta-analysis, the most efficacious psychological interventions for complicated grief are those based on cognitive behaviour therapy (CBT) principles (Wittouck et al., 2011); for example, Integrative CBT for Complicated Grief (Rosner et al., 2011); CBT for complicated grief (Boelen et al., 2013), and Complicated Grief Therapy (CGT; Shear & Shair, 2005). Only CGT specifically draws upon attachment theory (Shear & Shair, 2005); however, none of these models explicitly address how therapy could be adapted to take account of an individual’s attachment orientation.

The Dual Process Model of Coping may provide a useful framework when considering how to adapt psychological interventions regardless of the underlying approach (Stroebe & Schut, 1999). This model proposes that adaptive coping is comprised of oscillating between confronting and avoiding the loss. Confronting the loss (loss-oriented coping) may involve talking about the loss or experiencing painful emotions, whereas restoration-oriented coping may involve ‘taking time off’ from grief, getting back to previously enjoyed activities, developing new roles/identities. The Dual Process Model suggests that being stuck in grief may be due to an individual focusing heavily on either loss- or restoration-oriented coping, with a lack of balanced oscillation (Stroebe & Schut, 1999). Stroebe et al. (2005) suggest that an individual’s attachment orientation may underlie the ‘stuckness’ that characterises complicated grief. Stroebe et al. (2005) propose that individuals high in attachment anxiety may be focusing more on loss-oriented coping, and intervention may therefore be most beneficial if the bereaved person is supported to direct their attention towards restoration-focused tasks. Bereaved individuals high in attachment avoidance on the other hand, may well be suppressing loss-orientation needs, and thus therapy may specifically facilitate an individual to attend to the loss within the context of a supportive therapeutic relationship. Shear (2010) describes therapeutic, clinical approaches for managing avoidance when using the dual process model within an attachment-based perspective. However, there is a need for further empirical support to identify if tailoring grief interventions based on a person’s attachment orientation is effective.

## Conclusion

This review aimed to thoroughly examine and synthesise the evidence regarding attachment-related anxiety and avoidance and its relationship to complicated grief across a range of bereavement types and contexts. Attachment theory is a key paradigm for understanding differing responses to bereavement. In this systematic review, attachment anxiety and attachment avoidance were both consistently associated with higher levels of complicated grief, suggesting attachment insecurity may be an important vulnerability factor. This review therefore lends itself to providing guidance for clinicians and health professionals working in the field of bereavement. The existing evidence provides some pointers for clinicians working with bereaved individuals, who might find it helpful to explore the person’s attachment orientation, and formulate how attachment may help or hinder the person’s adjustment to their loss. The relationship between insecure attachment and complicated grief appears to exist across various bereavement contexts, and therefore is worth considering its clinical impact in all bereaved individuals irrespective of factors surrounding the death, or background of the individual. Further research is needed to explore the potential benefit of tailoring grief interventions to attachment orientations. Greater representation is needed from male populations and those of minority ethnic backgrounds, and more studies should utilise a longitudinal design. Initial findings regarding potential mediators and moderators require replication.

## Note

1 Participants from Meert et al.’s (2011) follow-up study have been excluded from the final count as participants were included in the original 2010 cohort.
